# Accelerated CCl_4_-Induced Liver Fibrosis in Hjv-/- Mice, Associated with an Oxidative Burst and Precocious Profibrogenic Gene Expression

**DOI:** 10.1371/journal.pone.0025138

**Published:** 2011-09-22

**Authors:** Giada Sebastiani, Kostas Gkouvatsos, Carmen Maffettone, Graziella Busatto, Maria Guido, Kostas Pantopoulos

**Affiliations:** 1 Lady Davis Institute for Medical Research, Jewish General Hospital, Montreal, Quebec, Canada; 2 Department of Medicine, McGill University, Montreal, Quebec, Canada; 3 Department of Diagnostic Sciences and Special Therapies, University of Padova, Padova, Italy; 4 Pathology Department, Azienda ULSS 15 Veneto Region, Italy; Yonsei University College of Medicine, Korea

## Abstract

Hereditary hemochromatosis is commonly associated with liver fibrosis. Likewise, hepatic iron overload secondary to chronic liver diseases aggravates liver injury. To uncover underlying molecular mechanisms, hemochromatotic hemojuvelin knockout (Hjv-/-) mice and wild type (wt) controls were intoxicated with CCl_4_. Hjv-/- mice developed earlier (by 2-4 weeks) and more acute liver damage, reflected in dramatic levels of serum transaminases and ferritin and the development of severe coagulative necrosis and fibrosis. These responses were associated with an oxidative burst and early upregulation of mRNAs encoding α1-(I)-collagen, the profibrogenic cytokines TGF-β1, endothelin-1 and PDGF and, notably, the iron-regulatory hormone hepcidin. Hence, CCl4-induced liver fibrogenesis was exacerbated and progressed precociously in Hjv−/− animals. Even though livers of naïve Hjv−/− mice were devoid of apparent pathology, they exhibited oxidative stress and immunoreactivity towards α-SMA antibodies, a marker of hepatic stellate cells activation. Furthermore, they expressed significantly higher (2–3 fold vs. wt, p<0.05) levels of α1-(I)-collagen, TGF-β1, endothelin-1 and PDGF mRNAs, indicative of early fibrogenesis. Our data suggest that hepatic iron overload in parenchymal cells promotes oxidative stress and triggers premature profibrogenic gene expression, contributing to accelerated onset and precipitous progression of liver fibrogenesis.

## Introduction

Disruption of iron homeostasis and accumulation of excess iron in tissues is associated with oxidative stress, cell injury and disease [1]. Hereditary hemochromatosis is characterized by chronic hyperabsorption and gradual deposition of iron within liver hepatocytes, while enterocytes and macrophages fail to retain iron due to inappropriately low expression of hepcidin [2], [3], [4]. This liver-derived circulating peptide controls iron fluxes by binding to and promoting degradation of the iron exporter ferroportin. Hepcidin is transcriptionally activated in response to iron-dependent and -independent stimuli by signaling via bone morphogenetic proteins (BMPs) or proinflammatory cytokines [5], [6], [7], [8]. The most frequent form of hereditary hemochromatosis is linked to mutations in HFE [9]. Juvenile hemochromatosis, an early onset variant, is mostly caused by mutations in hemojuvelin (Hjv) [10], a BMP co-receptor that is essential for signaling to hepcidin [11].

Development of liver disease is a common complication of hemochromatosis. Hepatic iron overload predisposes to fibrosis, cirrhosis and hepatocellular carcinoma [12], [13]. Moreover, the clinical phenotype associated with liver damage may be aggravated by comorbidities such as chronic viral hepatitis C, alcoholic liver disease and non-alcoholic steatohepatitis (NASH) [14], [15]. Interestingly, these non-hemochromatotic chronic liver diseases are highly prevalent in the general population and are often associated with mild to moderate secondary iron overload, which may exacerbate liver injury and contribute to hepatic fibrogenesis [16], [17].

The accumulation of liver fibrosis is a dynamic process characterized by deposition of collagen and other extracellular matrix proteins, following activation of quiescent hepatic stellate cells (HSCs) into a myofibroblast-like phenotype [18], [19], [20]. This results in secretion of several pro-fibrogenic cytokines, such as transforming growth factor beta 1 (TGF-β1), platelet-derived growth factor (PDGF), endothelin-1 and others. Progression of liver fibrosis towards end-stage liver disease depends on many cofactors, including hepatic iron load [12], [13], [16], [17]. Nevertheless, even though the toxicity of iron is generally attributed to oxidative stress, its exact role in the pathway of liver fibrogenesis remains unclear.

Rodent models of liver fibrosis recapitulate key aspects of the pathogenic mechanisms [21], [22]. Treatment with carbon tetrachloride (CCl_4_), a known hepatotoxin, represents an established approach to trigger liver fibrogenesis, which is relatively well characterized for histological, biochemical and molecular alterations. Iron intoxication, achieved by feeding of animals with carbonyl iron, was found to act synergistically with CCl_4_ (or alcohol) for development of liver damage in most [23], [24], [25], [26] but not all cases [27], [28]. Interestingly, it is believed that unlike in humans, iron overload per se does not suffice to cause liver fibrosis in rodents, with the notable exception of gerbils [29], [30].

To decipher the role of iron in the development of liver fibrosis, we employed here Hjv−/− mice as a genetic model of severe iron overload. We show that excessive hepatic iron deposition potentiates chemically-induced liver fibrogenesis by promoting an oxidative burst and premature induction of profibrogenic cytokines. Moreover, we demonstrate that naïve Hjv−/− animals manifest early signs of fibrogenesis and liver disease.

## Results

### Hjv−/− mice exhibit accelerated liver damage in response to CCl_4_ intoxication

Mice with targeted disruption of Hjv spontaneously develop iron overload due to defective iron sensing and excessive absorption of dietary iron, constituting an animal model of juvenile hemochromatosis [31], [32]. To assess the effects of iron overload in liver fibrogenesis, Hjv−/− and isogenic wt animals were subjected to treatment with CCl_4_ (or corn oil vehicle as control) over a period of up to 6 weeks. We noticed that mice injected with corn oil were phenotypically indistinguishable from untreated ones; therefore, unless otherwise indicated, “baseline” values represent an average from untreated and corn oil-treated animals.

**Figure 1:**
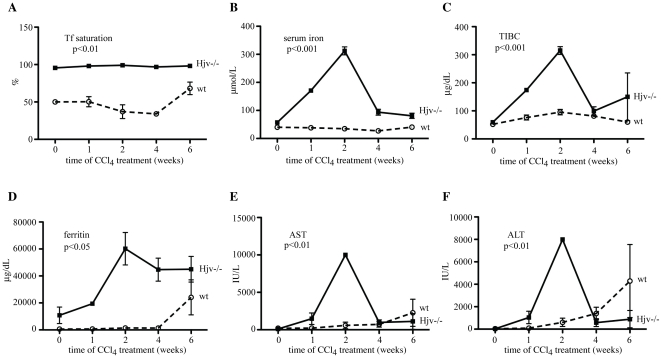
Hjv−/− mice exhibit early and dramatic increases in serum iron indices and transaminases, following CCl_4_-mediated liver injury. (A–C) Transferrin saturation, serum iron and TIBC, respectively. (D–F) Levels of serum ferritin and the transaminases AST and ALT, respectively. The p values refer to Hjv−/− vs wt and were obtained by the ANOVA test.

As expected, untreated Hjv−/− mice exhibited very high serum iron indices as compared to wt counterparts (transferrin saturation: 93.8±4.3 vs 50.8±7.5%, p<0.0001; serum iron: 57.2±8.6 vs 36.5±3.6 µmol/L, p<0.0001; serum ferritin: 10,785.7±6,120.5 vs 595±77 µg/dL, p<0.0001), consistently with an iron overload phenotype. Serum iron parameters were subsequently analyzed at different time intervals. Tranferrin saturation remained largely unaffected by CCl_4_ in Hjv−/− mice and appeared to slightly fluctuate and increase towards the last phase of the treatment in wt animals (Fig. 1A). Interestingly, CCl_4_ triggered a sharp ∼5-fold increase in serum iron levels of Hjv−/− mice that peaked within 2 weeks and decreased afterwards (Fig. 1B). This was accompanied by a commensurate ∼5-fold expansion of total iron binding capacity (TIBC; Fig. 1C), indicating that excess of serum iron was shielded by transferrin. The CCl_4_ treatment did not significantly affect serum iron and TIBC in wt animals.

By the second week of CCl_4_ treatment, serum ferritin increased dramatically in Hjv−/− mice to almost 60,000 µg/dL, possibly as a result of inflammation (see also Fig. S3A), and remained extremely elevated until the sixth week (Fig. 1D). By contrast, in wt mice, very high ferritin values (up to ∼20,000 µg/dL) were only recorded at the sixth week of CCl_4_ treatment. Analogous differential responses between Hjv−/− and wt mice were observed regarding the CCl_4_-dependent induction of serum aspartate aminotransferase (AST) and alanine aminotransferase (ALT). Thus, Hjv−/− mice exhibited remarkably augmented AST and ALT values during the second week of CCl_4_ treatment (to ∼10,000 and 8,000 IU/L, respectively), while delayed and comparatively less dramatic responses were observed in wt animals (Figs. 1E and F). These data suggest that CCl_4_ intoxication causes accelerated and more profound liver damage to Hjv−/− mice, as compared to wt controls.

### Hepatic iron overload potentiates the development of chemically-induced liver fibrosis

**Figure 2:**
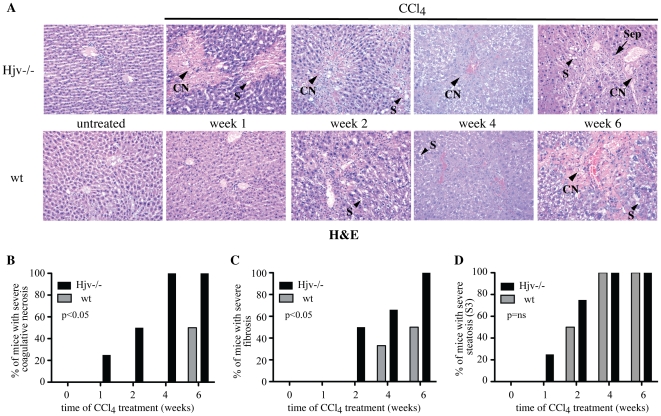
Hjv−/− mice develop earlier and more severe liver disease in response to CCl_4_ intoxication. (A) H&E staining of liver sections from Hjv−/− and wt mice subjected to treatment with CCl_4_ for 1–6 weeks. Controls (ctrl) correspond to livers from untreated Hjv−/− and wt mice, which exhibit identical tissue architecture with respective corn oil-treated counterparts. Representative areas with coagulative necrosis (CN), steatosis (S) and septa (Sep) are indicated by arrowheads. Original magnification 20x. (B–D) Semiquantitative grading of hepatic coagulative necrosis (B) fibrosis (C) and steatosis (D); data are compiled from n = 32 mice. The p values refer to Hjv−/− vs wt and were obtained by the ANOVA test; ns = non-significant.

Histological analysis with hematoxylin and eosin (H&E) revealed advanced coagulative necrosis in liver sections of Hjv−/− mice already one week following CCl_4_ treatment (Fig. 2A and B). Neither Hjv−/− nor wt livers displayed any immunoreactivity with caspase-3, a cell death protease and marker of apoptosis. This seems to be in agreement with the morphology of cell death in this model of hepatotoxicity, which mainly shows features of coagulative necrosis. Hepatic damage developed more gradually in wt animals and reached severe grades only by the end of the treatment. Within six weeks of hepatotoxin administration, all Hjv−/− but half of the wt mice manifested severe fibrosis (Figs. 2A and C). CCl_4_-treated animals of both genotypes progressively developed severe (grade S3) steatosis (Figs. 2A and D).

**Figure 3:**
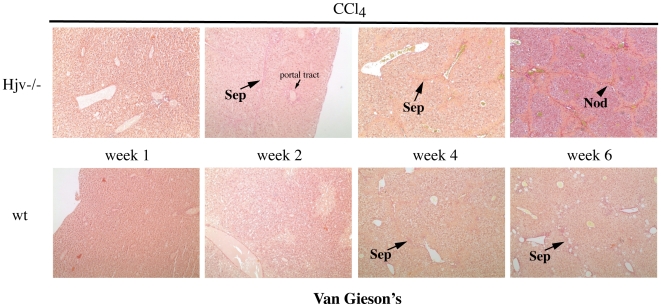
Assessment of fibrillar collagen. Liver sections from CCl_4_-treated Hjv−/− and wt mice were stained with van Gieson's. Septa (Sep) and nodules (Nod) are indicated by arrows. Original magnification 20x.

The increased susceptibility of CCl_4_-treated Hjv−/− mice to fibrogenesis was validated by visualization of fibrillar collagen upon van Gieson's staining (Fig. 3). Thus, livers of Hjv−/− mice developed rare thin septa at the second week of CCl_4_ treatment, and numerous septa with architectural alteration and nodules afterwards. By contrast, in livers of wt mice, rare thin septa were only visible in the context of necrotic areas during the fourth and sixth week of CCl_4_ treatment. HSC activation was assessed by immunohistochemical staining of liver sections (Fig. S1A) and by Western blotting of liver lysates (Fig. S1B) with an α-SMA antibody; expression of this marker increased in response to CCl_4_ treatment. Semiquantitative analysis of the amount of positive stained area in the immunohistochemical experiment shows a time-dependent increase in Hjv−/− and to a lesser extent in wt animals. Activated HSCs were mainly observed at the site of damage, consistently with literature [20]. Nevertheless, densitometric quantification of the Western blot did not reveal any statistically significant time-dependent alterations in α-SMA expression among Hjv−/− and wt mice, likely attributable to low sensitivity of the assay. A masking effect due to contribution of further cell types to the generation of α-SMA expressing activated hepatic myofibroblasts [20] is also possible.

Staining with Perls' Prussian blue confirmed the hepatic iron overload phenotype of Hjv−/− mice, with iron deposits primarily detectable in parenchymal cells (Fig. S2A), in agreement with published data [31], [32]. The CCl_4_ treatment promoted sequestration of iron in Kupffer cells, a known inflammatory response [33]. In quantitative terms (Fig. S2B), livers from Hjv−/− mice contained ∼10 times more iron as compared to wt counterparts (5,769.7±1,760.1 vs 500.4±211.4 µg of Fe per g of dry tissue, p<0.001). Apparently, hepatic iron loading was not significantly affected by CCl_4_.

**Figure 4:**
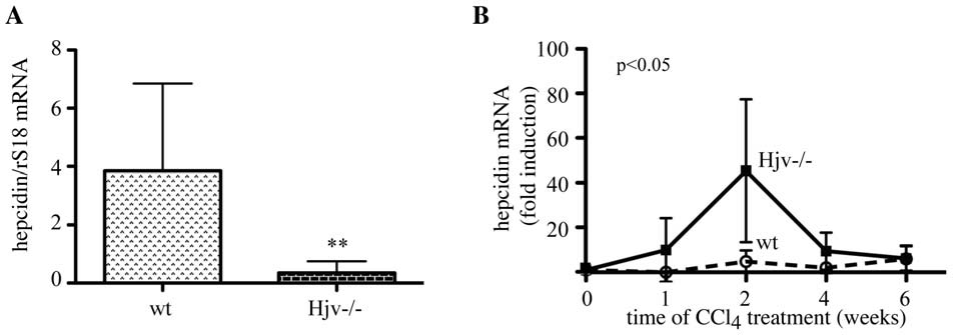
CCl_4_ intoxication promotes profound transient upregulation of hepcidin mRNA in Hjv−/− mice. (A) Comparison of basal hepcidin mRNA levels between Hjv−/− and wt mice. (B) Time-dependent effects of CCl_4_ treatment on hepcidin mRNA expression. The indicated induction corresponds to the ratio of hepcidin mRNA values obtained from livers of CCl_4_- and corn oil-treated animals. The p values refer to Hjv−/− vs wt and were obtained by the ANOVA test. ** p<0.01 vs wt (Student's t test).

As expected [31], [32], Hjv−/− mice express pathologically low levels of hepcidin mRNA (∼11 times less than wt, p<0.001; Fig. 4A), in line with the function of Hjv as a BMP co-receptor that is necessary for iron-dependent signaling to hepcidin [11]. Interestingly, Hjv−/− mice manifested a transient dramatic increase in hepcidin mRNA expression during the second week of CCl_4_ treatment (compared to Hjv−/− mice treated with corn oil), while an analogous response was modest in wt animals (Fig. 4B, p<0.05). We conclude that hepatic iron overload accelerates and exacerbates liver damage caused by CCl_4_.

### Hepatic iron and CCl_4_ synergistically promote early induction of profibrogenic genes, associated with an oxidative burst

**Figure 5:**
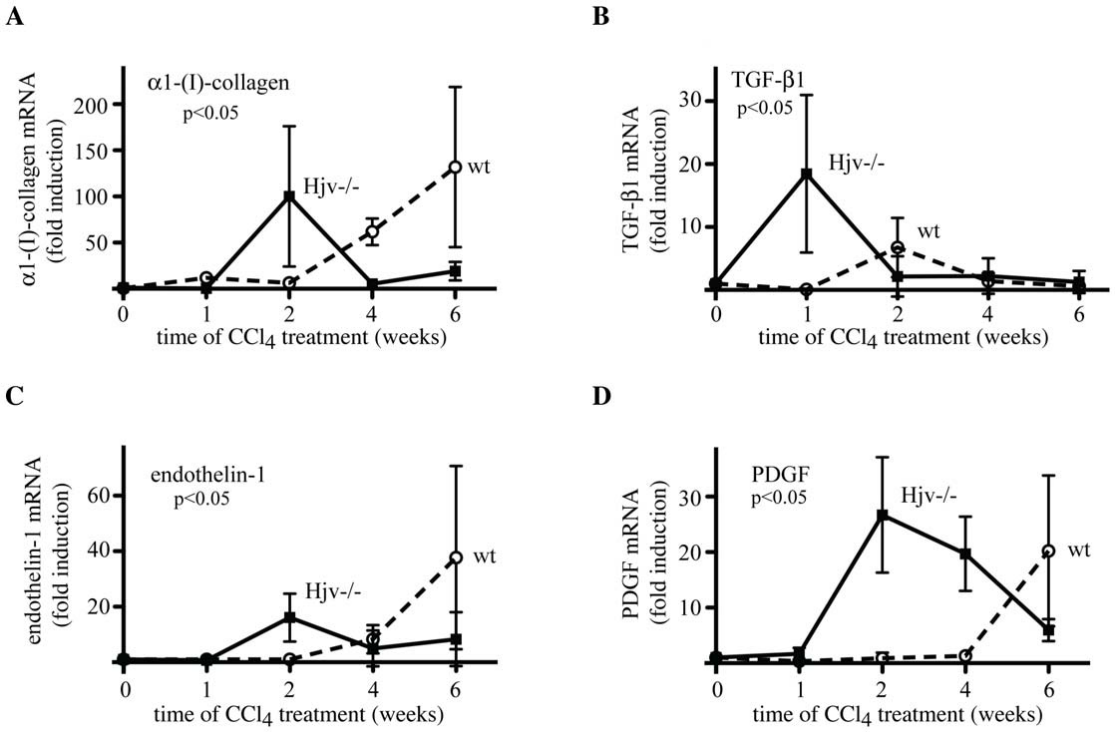
Precocious expression of profibrogenic molecules in livers of Hjv−/− mice, following treatment with CCl_4_. The induction of the mRNAs encoding α1-(I)-collagen (A), TGF-β1 (B), endothelin-1 (C) and PDGF (D) corresponds to the ratios of respective mRNA values obtained from CCl_4_- and corn oil-treated animals. The p values refer to Hjv−/− vs wt and were obtained by the ANOVA test.

To unravel the molecular mechanisms by which iron aggravates CCl_4_-induced hepatotoxicity, mouse livers were analyzed for expression of genes that are implicated in fibrogenetic pathways. The exposure of wt mice to CCl_4_ resulted in a potent (up to ∼125-fold) activation of α1-(I)-collagen mRNA (as compared to oil-treated animals), which was evident after the fourth week and peaked at the sixth week of treatment (Fig. 5A). In Hjv−/− mice, CCl_4_ triggered an earlier induction (∼100-fold) of α1-(I)-collagen mRNA within 2 weeks; notably, α1-(I)-collagen mRNA levels were almost normalized afterwards.

**Figure 6:**
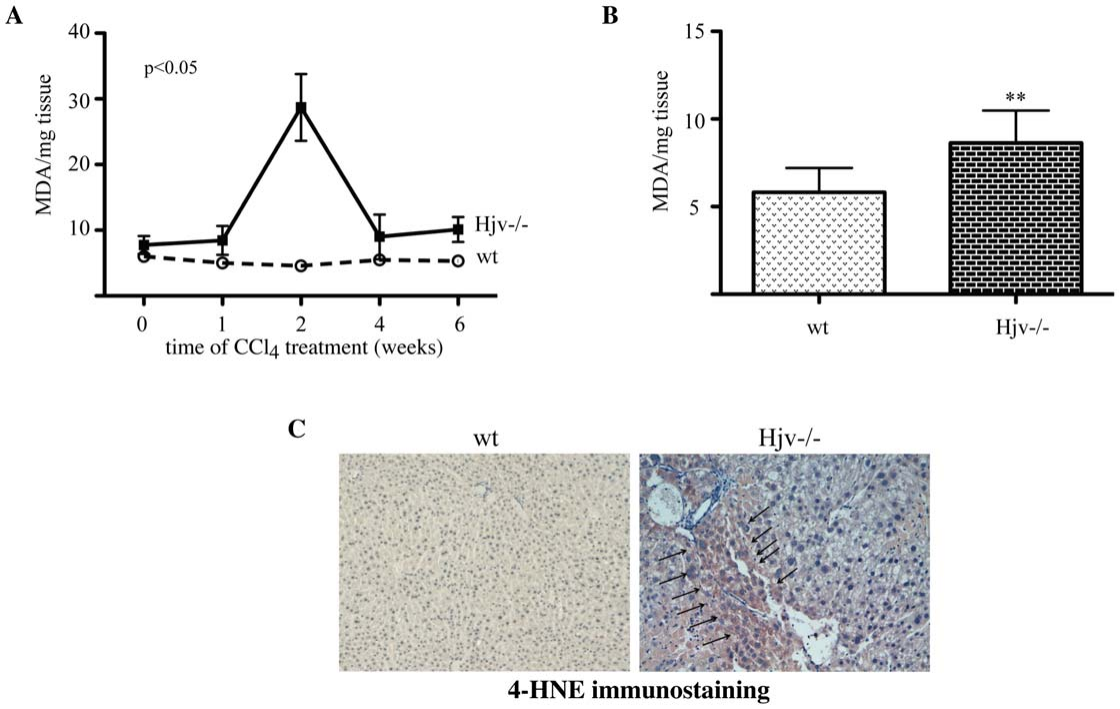
Oxidative stress and HSC activation in livers of naïve Hjv−/− mice. (A) Evidence for a transient oxidative burst in livers of Hjv−/− mice, following CCl_4_ intoxication. Hepatic MDA was quantified by the TBARS assay. The p value refers to Hjv−/− vs wt and was obtained by the ANOVA test. (B) Measurement of hepatic MDA levels in naïve untreated Hjv−/− and wt mice; data are compiled from n = 6 mice in each group. (C) Immunohistochemical detection of 4-HNE (arrows) in livers from naïve untreated Hjv−/− mice; data from one representative Hjv−/− and wt mouse are shown. Original magnification 20x. ** p<0.01 vs wt (Student's t test).

Similar results were obtained by analyzing the expression of profibrogenic cytokines (Figs. 5B-D). Hence, in wt mice, the CCl_4_ treatment caused an induction of the mRNAs encoding TGF-β1 (∼6-fold within 2 weeks), endothelin-1 and PDGF (∼38- and ∼20-fold, respectively, with a peak at 6 weeks). By contrast, in Hjv−/− animals, CCl_4_ elicited accelerated induction of TGF-β1 mRNA within 1 week (∼19-fold), as well as endothelin-1 and PDGF mRNAs within 2 weeks (∼32- and ∼20-fold, respectively). Importantly, livers of Hjv−/− mice manifested an oxidative burst during the second week of CCl_4_ treatment, as judged by the ∼3-fold increase in levels of malondialdehyde (MDA), a product of lipid peroxidation (Fig. 6A). This was also associated with a peak in TNF-α mRNA expression, which was ∼6.7-fold stronger (p<0.05) in Hjv−/− animals (Fig. S3A), while IL-1β mRNA levels were slightly augmented after the second week of CCl_4_ treatment in all mice (Fig. S3B). Taken together, these findings highlight a positive synergy of hepatic iron overload in chemically-induced liver fibrogenesis, possibly via an oxidative stress-dependent pathway, that culminates in precocious activation of profibrogenic gene expression and upregulation of TNF-α.

### Naïve Hjv−/− mice exhibit hepatic oxidative stress and present early signs of liver fibrogenesis

The data in Fig. 6A show a tendency for increased MDA expression in livers of Hjv−/− mice as compared to wt controls, even without CCl_4_ treatment (week = 0). To validate this, we further analyzed MDA levels in livers of naïve untreated Hjv−/− and wt mice. The former exhibited ∼50% higher MDA levels compared to wt (Fig. 6B, p<0.01), indicative of oxidative stress. Furthermore, Hjv−/− livers allowed immunohistochemical detection of 4-hydroxy-2-nonenal (4-HNE), another lipid peroxidation product (Fig. 6C). As expected, positive 4-HNE staining was also evident in livers of all CCl_4_-animals (Fig. S4).

**Figure 7:**
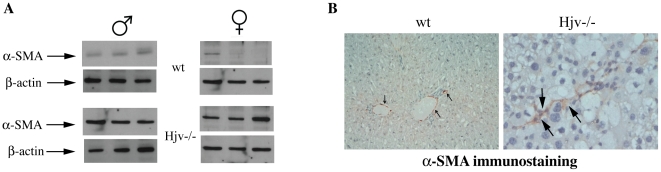
Evidence for HSC activation in naïve Hjv−/− mice. (A) Liver extracts of untreated wt and Hjv−/− mice (three male and three female, each) were analyzed by Western blotting with antibodies against α-SMA and housekeeping β-actin. Hjv−/− mice exhibit marked expression of hepatic α-SMA (bottom). (B) Immunohistochemical staining with an α-SMA antibody in liver sections of wt and Hjv−/− mice. Only livers of naïve untreated Hjv−/− mice (right) manifest α-SMA-positive sinusoidal cells (arrows). The small arrows in wt liver section (left) indicate positive internal control represented by normal portal vessels. Original magnification 20x for wt and 40x for Hjv−/− liver sections.

We noticed that livers of oil-treated Hjv−/− mice expressed relatively high levels of α-SMA (Fig. S1B, left panel). We further analyzed the expression of α-SMA in naïve untreated Hjv−/− and wt mice by Western blotting and immunohistochemistry. Only livers of naïve untreated Hjv−/− animals had an elevated α-SMA content (Fig. 7A), associated with the presence of α-SMA-positive sinusoidal cells (Fig. 7B), demonstrating HSC activation in the absence of any chemical hepatotoxin.

**Figure 8:**
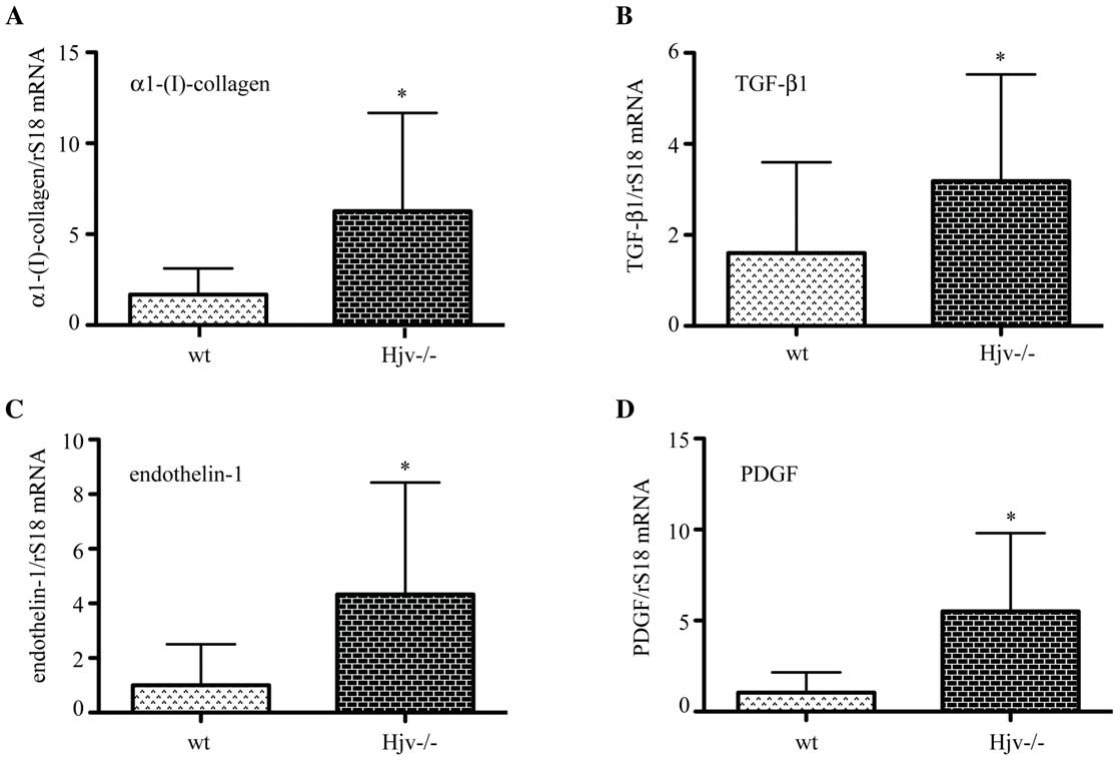
Naïve Hjv−/− mice exhibit increased expression of hepatic profibrogenic molecules. Comparative expression of the mRNAs encoding α1-(I)-collagen (A), TGF-β1 (B), endothelin-1 (C) and PDGF (D) in livers from wt and Hjv−/− mice. *p<0.05 vs wt (Student's t test).

A comparison of hepatic expression profiles of profibrogenic genes uncovered higher levels of the mRNAs encoding α1-(I)-collagen (∼3.7-fold, p<0.05), TGF-β1 (∼2-fold, p<0.05), endothelin-1 (∼4-fold, p<0.05) and PDGF (∼5.5-fold, p<0.05) in naïve Hjv-/- mice, as compared to wt counterparts (Fig. 8). No significant differences in the expression of the proinflammatory cytokines TNF-α and IL-1β were found among the two genotypes (Figs. S3C and S3D). In conclusion, these data suggest that hepatic iron overload in Hjv−/− animals promotes in its own right oxidative stress, activation of HSCs and profibrogenic gene expression. Such responses are consistent with early signs of liver fibrogenesis, prior to the development of fibrosis.

## Discussion

We show here that hemochromatotic Hjv−/− mice are extremely vulnerable to the hepatotoxicity of CCl_4_ and develop premature, and more profound biochemical and histological symptoms of liver disease compared to wt counterparts. Within only two weeks of CCl_4_ treatment, Hjv−/− mice manifested dramatic values of serum transaminases and ferritin, as well as severe hepatic coagulative necrosis (Figs. 1 and 2), which very likely accounts for the ∼5-fold increase in serum iron levels due to release of the metal from necrotic cells. The parallel elevated TIBC can be explained by induction of transferrin expression, via an unknown protective mechanism, which presumably serves to prevent accumulation of circulating redox-active and potentially toxic non-transferrin-bound iron (NTBI) [34]. Nevertheless, the marked increase in hepatic MDA levels (Fig. 6A) strongly suggests the presence of an at least transient pool of redox-active iron, that promotes lipid peroxidation. The absence of caspase 3 immunoreactivity is consistent with necrotic cell death. Nevertheless, we cannot exclude that only small amount of caspase-3 (below the level of detectability) is required or that a caspase-3-independent form of programmed cell death may be involved.

Contrary to Hjv−/− mice, wt controls mounted a delayed and modest increase of serum transaminases and ferritin (but not iron) after 4 weeks of CCl_4_ treatment, as a result of gradual necroinflammatory activity. Importantly, Hjv−/− mice eventually developed more advanced fibrosis during the CCl_4_ treatment (Figs. 2 and 3). Considering that inflammatory stimuli are known to diminish hepatic Hjv expression in wt mice [32], [35], it is unlikely that the enhanced sensitivity of Hjv−/− mice to chemically-induced liver fibrosis is caused by an altered inflammatory response. Overall, our data strongly suggest that the severe necrotic damage in livers of Hjv−/− animals is the consequence of iron-dependent oxidative stress.

Excessive hepatic iron has been recognized for years as a factor contributing to the development of liver fibrosis [12], [13], [16], [17]. However, progress in understanding the underlying pathogenic mechanisms has been hampered by the lack of appropriate animal models. We employ here for the first time a genetic mouse model of hemochromatosis to investigate the role of iron in the initiation of liver fibrogenesis. Previous approaches involved simultaneous intoxication of rodents with carbonyl iron and a chemical hepatotoxin (CCl_4_ or alcohol). While most of the earlier studies concluded that carbonyl iron enhanced the liver-damaging effects of chemical hepatotoxins [23], [24], [25], [26], feeding rats with carbonyl iron failed to potentiate alcohol-mediated liver fibrosis [28] and has also been reported to protect rats from CCl_4_-dependent hepatic fibrogenesis and carcinogenesis [27]. Such discrepancies may be related to temporal differences in the effects of iron. Our study is the first one that addresses the role of iron in chemically-induced liver fibrogenesis in a time-dependent manner. The data presented here clearly demonstrate that iron elicits early profibrogenic events, that may escape attention if the experimental design is not appropriate. Previous discrepancies may also be related to variabilities in the distribution of iron deposits among hepatocytes and reticuloendothelial cells in carbonyl iron intoxication models [36], considering that the iron content of macrophages is crucial for the production of proinflammatory cytokines via NF-κΒ [37], [38] and for further immune effector functions [39].

Hjv−/− mice faithfully reproduce phenotypic hallmarks of hemochromatosis, such as excessive dietary absorption and deposition of iron in liver parenchymal cells, while macrophages remain iron-deficient. Therefore, this model is more pertinent to address the role of hereditary iron overload in early liver fibrogenesis. Moreover, elucidating pathophysiological and molecular responses of Hjv−/− mice to chemical hepatotoxins may deserve particular attention in the context of the increased liver damage documented in hemochromatosis patients when another cause of hepatic chronic injury is present; for example, chronic hepatitis C, alcoholic liver disease or NASH [14], [15]. The eventual withholding of iron in macrophages of CCl_4_-mice is congruous with the pathology of chronic liver diseases, further indicating the physiological relevance of the model described here.

The data in Fig. 5 suggest that hepatic iron overload accelerates the CCl_4_-mediated induction of profibrogenic molecules such as α1-(I)-collagen, TGF-β1, endothelin-1 and PDGF, in a synergistic fashion. A key molecular event with a possible causative role appears to be the robust precocious activation of TGF-β1 mRNA expression during the first week of CCl_4_-treatment. In wt mice this response is not only delayed (by one week), but also considerably weaker. In previous experiments, the conditional tetracycline-inducible overexpression of TGF-β1 sufficed to promote fibrosis in mice, which was regressed upon switching off transgenic production of this cytokine [40], suggesting that inhibition of iron-dependent TGF-β1 activation may mitigate the profibrogenic effects of iron. Interestingly, the early induction of TGF-β1 mRNA in CCl_4_-treated Hjv−/− mice did not last more than one week. During the second week of CCl_4_ treatment, the expression of α1-(I)-collagen, endothelin-1 and PDGF was likewise transiently augmented and this correlated with an apparent oxidative burst (Fig. 6A). The kinetics of α1-(I)-collagen, endothelin-1 and PDGF mRNA expression exhibit striking similarities with the kinetic profile of MDA accumulation, suggesting a potential mechanistic link between profibrogenic gene expression and oxidative stress.

TGF-β1 and α1-(I)-collagen mRNAs were previously reported to be synergistically upregulated in livers of rats fed with carbonyl iron and ethanol [25]. Signals for the early potent induction of TGF-β1 by activated HSCs in Hjv−/−mice may originate from iron-laden hepatocytes and/or from Kupffer cells. The iron content of the latter appears to increase substantially during the CCl_4_ treatment, consistently with the noticeable upregulation of hepcidin mRNA (Fig. 4B), which is responsive to inflammatory stimuli in Hjv−/− mice [32]. Even though this temporary effect did not have an impact in overall serum and histological iron indices, it is likely to modulate cytokine-induced signaling networks in Kupffer cells [41]. Tissue ferritin is capable of activating IL-1β expression [42], which in turn is known to stimulate hepcidin mRNA transcription [43]; nevertheless, the modest induction of IL-1β mRNA (Fig. S3B) appears to exclude any association between the dramatic increase in serum ferritin levels (Fig. 1D) with the hepcidin upregulation (Fig. 4B) in CCl_4_-treated Hjv−/− mice.

Another major finding of this work is that naïve Hjv−/− mice express significantly higher basal levels of profibrogenic molecules (α1-(I)-collagen, TGF-β1, endothelin-1 and PDGF) compared to wt animals (Fig. 8). Furthermore, livers of naïve Hjv−/− mice contain activated HSCs (Fig. 7) and manifest signs of lipid peroxidation (Fig. 6). Collectively, these data suggest that, despite the absence of obvious hepatic fibrosis, Hjv−/− mice initiate spontaneously fibrogenetic pathways, possibly as a result of iron-dependent oxidative stress.

It should be noted that iron-loaded livers of hemochromatosis patients were also found to exhibit oxidative stress and enhanced expression of TGF-β1 [44], as well as to contain activated HSCs prior to the development of histological fibrosis [45]. Likewise, iron-dependent oxidative stress [46] and increase in α1-(I)-collagen mRNA expression [47], [48] has been documented in rats. Nevertheless, neither intoxication of rats with carbonyl iron [36], [49], nor iron overload of mice with genetically disrupted iron homeostasis [31], [50] appear to cause significant hepatic histological alterations and liver disease. Among rodents, only gerbils were reported to develop severe iron-dependent liver damage, following repeated parenteral injections with iron dextran, which could be inhibited by the antioxidant vitamin E [29], [30]. Along these lines, due to the absence of significant hepatic fibrosis in Hjv−/− mice it has been hypothesized that mice “may be protected from the toxic effects of iron overload” [31]. However, the data presented here document a spontaneous early activation of hepatic fibrogenesis in hemochromatotic Hjv−/− mice, that may require more time or a “second hit” to progress into full-blown liver disease.

## Materials and Methods

### Animals

All experimental procedures were approved by the Animal Care Committee of McGill University (protocol 4966). Hjv−/− mice, maintained on an inbred 129S6/SvEvTac background [31], were kindly provided by Dr. Nancy Andrews (Duke University). Isogenic wild type (wt) mice were purchased from the Charles River Laboratories (Cambridge, MA). All animals were housed in macrolone cages (up to 5 mice/cage, 12∶12 h light-dark cycle: 7 am–7 pm; 22±1°C, 60±5% humidity) according to standard institutional guidelines, and had free access to water and food.

### Induction of liver injury by CCl_4_ intoxication

6-week old Hjv−/− and wt mice were subjected to treatment with CCl_4_ to induce liver injury, or with sterilized corn oil vehicle as control. The animals were divided into 4 experimental groups (n = 16 mice for each group): a) Hjv−/− mice treated with CCl_4_; b) Hjv−/− mice treated with corn oil; c) wt mice treated with CCl_4_; and d) wt mice treated with corn oil. CCl_4_ (2 ml per kg of animal, in a 10% solution of corn oil) or corn oil were injected intraperitoneally twice per week for up to 6 weeks. Four mice of each group were sacrificed at weeks 1, 2, 4 and 6 by cervical dislocation. Age-matched Hjv−/− and wt mice were sacrificed without any previous treatment, to obtain baseline experimental variables. Before sacrifice, the mice were anesthetized and exsanguinated by cardiac puncture to obtain blood serum.

### Serum biochemistry

Transferrin saturation, TIBC and levels of serum iron, ferritin, AST and ALT were measured by a Roche Hitachi 917 Chemistry Analyzer at the Biochemistry department of the Jewish General Hospital.

### Preparation of liver samples

Livers were washed with ice-cold phosphate buffered saline (PBS) and dissected into smaller pieces. Aliquots were snap frozen at liquid nitrogen and stored at −80°C, or fixed in 10% buffered formalin and embedded in paraffin.

### Quantification of non-heme iron

Hepatic non-heme iron was measured by the ferrozine assay [51]. Results are expressed as micrograms of iron per gram of dry tissue weight.

### Histological analysis

Deparaffinized liver sections were stained with H&E and with trichrome stain for collagen. Ferric iron deposits were visualized by Perls' Prussian blue with the Accustain® Iron Stain kit (Sigma). The slides were evaluated by an experienced pathologist who was unaware of any experimental information. CCl_4_-mediated coagulative necrosis was graded as mild, when involving only a rim of perivenular zone 3 hepatocytes and severe when more extensive, confluent, necrosis was observed involving zone 2 and 1 [52]. Fibrosis was scored as mild (portal-periportal or intralobular without septa formation) or severe (fibrous septa with or without cirrhosis) [52]. Steatosis (S) was separately assessed on a four-grade scale, according to the percentage of hepatocytes with fat [53] (grade S0, absent; grade S1, <10% hepatocytes; grade S2, 10–30%; grade S3, >30%).

### Immunohistochemistry

Following deparaffinization, incubation in methanolic H_2_O and standard microwave treatment, sections were incubated overnight at 4°C with mouse monoclonal antibodies against α-SMA (Biosensis; 1∶150 dilution), 4-HNE (Alpha Diagnostic; 1∶200 dilution) or caspase 3 (Ab-4 from Calbiochem; 1∶40 dilution). These primary antibodies were previously biotinylated by using the DAKO™ ARK (animal research kit). As control, the primary antibodies were replaced by normal mouse serum. After wash with PBS, the slides were incubated for 15 minutes with streptavidin-peroxidase. Staining was completed by incubation with 3,3′-diaminobenzidine (DAB) for 5 minutes, which results in a brown-colored precipitate at the antigen site. For quantification of α-SMA, the amount of positive stained area was determined using an arbitrary semiquantitative score from 0 to 4: 0, no staining 1, mild-scatter positive staining; 2, moderate staining; 3, marked staining; and 4, intense staining through the liver parenchyma.

### TBARS assay

For the quantification of MDA, frozen liver samples were disrupted in cold PBS by means of a disposable pestle (Axygen Inc). The whole homogenate was used for the Thiobarbituric Acid Reactive Substances Assay (OXItek TBARS Assay Kit, Zeptometrix). Results are expressed as nmol of MDA per mg of tissue weight.

### Western blotting

Frozen liver aliquots were suspended in a lysis buffer containing 20 mM Tris-Cl pH 7.4, 40 mM KCl, 1% Triton X-100, an EDTA-free protease inhibitor cocktail (Roche) and a Halt phosphatase inhibitor Cocktail (Thermo Scientific), and homogenized with a TissueRuptor handheld homogenizer (Qiagen). Cell debris was cleared by centrifugation and the protein concentration was measured with the Bradford reagent (BioRad). Protein extracts (30 µg) were resolved by SDS-PAGE on 10% gels and the proteins were transferred onto nitrocellulose filters (BioRad). The blots were saturated with 10% non-fat milk in PBS containing 0.1% (v/v) Tween-20 (PBS-T) and probed with a 1:100 diluted monoclonal antibody against α-SMA (Sigma). After three washes with PBS-T, the blots were incubated with 1:5000 diluted peroxidase-coupled rabbit anti-mouse IgG (Sigma). The peroxidase signal was detected by enhanced chemiluminescence with the Western Lightning ECL kit (Perkin Elmer).

### Quantitative real-time PCR (qPCR)

Total RNA was isolated from frozen liver tissue using the RNeasy Midi kit (Qiagen); its quality was assessed by determining the 260/280 nm absorbance ratios and by agarose gel electrophoresis. qPCR was performed as previously described [54] by using gene-specific primers (Table 1). Ribosomal protein S18 (rS18) was used as housekeeping gene for normalization.

**Table 1:** Sequence of oligonucleotide primers used in real-time qPCR reactions.

Mouse gene	Accession number	Forward primer sequence	Reverse primer sequence
r18S	NR_003278	GAATAATGGAATAGGACCGCGG	GGAACTACGACGGTATCTGATC
Hamp (hepcidin)	NM_032541	AAGCAGGGCAGACATTGCGAT	CAGGATGTGGCTCTAGGCTATGT
α1-(I)-collagen	NM_007742	CCAAGGGTAACAGCGGTGAA	CCTCGTTTTCCTTCTTCTCCG
TGF-β1	NM_011577	GGTTCATGTCATGGATGGTGC	TGACGTCACTGGAGTTGTACGG
endothelin-1	NM_010104	GAAACAGCTGTCTTGGGAGC	AGTTCTTTTCCTGCTTGGCA
PDGF-D	NM_027924	ACTCTCACTGCTGATGCCCT	GACTGCATTGGTCAGCTTCA

### Statistical analysis

Quantitative data were expressed as mean ± standard deviation (SD). Statistical analysis was performed by using the two-tailed Student's t test or the one way ANOVA test, with the GraphPad Prism software (v. 5.0c). A probability value p<0.05 was considered to be statistically significant.

## Supporting Information

Figure S1 **Activation of HSCs in response to CCl_4_ treatment.** (A) Immunohistochemical detection of α-SMA (arrows) in liver sections from Hjv−/− (top) and wt (bottom) mice. Original magnification 20x, except wt mice week 2 (40x). (B) Western blot analysis of α-SMA and housekeeping β-actin in liver extracts.
(TIF)

Figure S2 **Hjv−/− mice exhibit severe iron overload in parenchymal cells, while the CCl_4_ treatment promotes iron retention within Kupffer cells.** (A) Perl' s staining of liver sections from Hjv−/− and wt mice subjected to treatment with CCl_4_ for 1–6 weeks. Iron-loaded Kupffer cells are indicated with arrowheads. Original magnification 20x. (B) Quantification of hepatic non-heme iron by the ferrozine assay. *p<0.05 vs wt (Student's t test).
(TIF)

Figure S3 **Expression of proinflammatory cytokines in Hjv−/− and wt mice.** (A and B) The induction of TNF-α and IL-1β mRNAs corresponds to the ratios of respective values obtained from CCl_4_- and corn oil-treated animals. The p values refer to Hjv−/− vs wt and were obtained by the ANOVA test; ns = non-significant. (C and D) Comparative expression of TNF-α and IL-1β mRNAs in livers from naïve wt and Hjv−/− mice.
(TIF)

Figure S4 **Immunohistochemical detection of 4-HNE (arrows) in livers of CCl_4_-treated Hjv-/- and wt mice.** Original magnification 20x, except wt mice week 4 (40x).
(TIF)
