# Supplementary figures and images for: Accelerated CCl4-Induced Liver Fibrosis in Hjv-/- Mice, Associated with an Oxidative Burst and Precocious Profibrogenic Gene Expression
Source: PLoS One. 2011 Sep 22;6(9):e25138. doi: 10.1371/journal.pone.0025138 (PMC3178612; doi:10.1371/journal.pone.0025138)

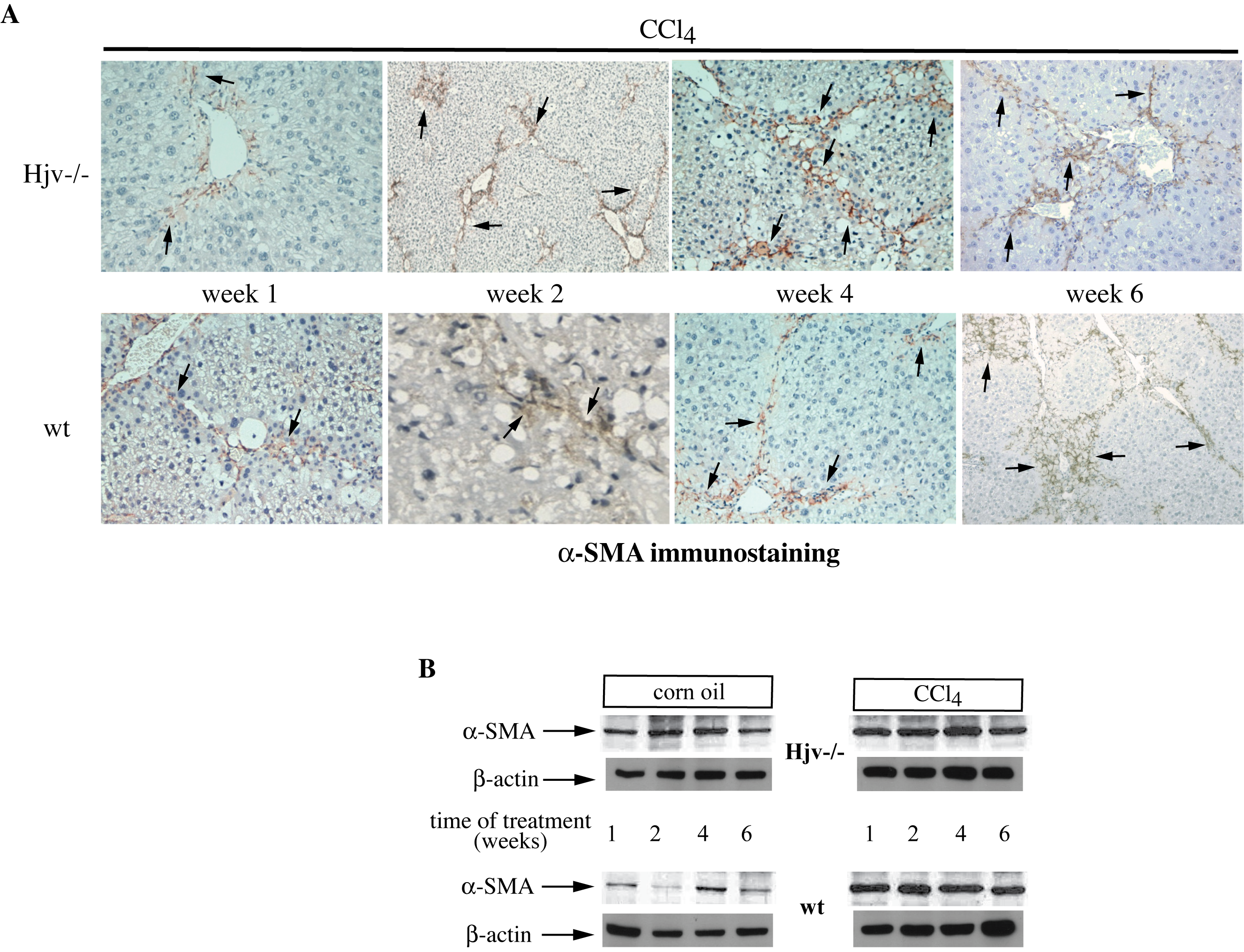

Supplement: Figure S1 — Activation of HSCs in response to CCl4 treatment. (A) Immunohistochemical detection of α-SMA (arrows) in liver sections from Hjv−/− (top) and wt (bottom) mice. Original magnification 20x, except wt mice week 2 (40x). (B) Western blot analysis of α-SMA and housekeeping β-actin in liver extracts. (TIF) [file pone.0025138.s001.tif]

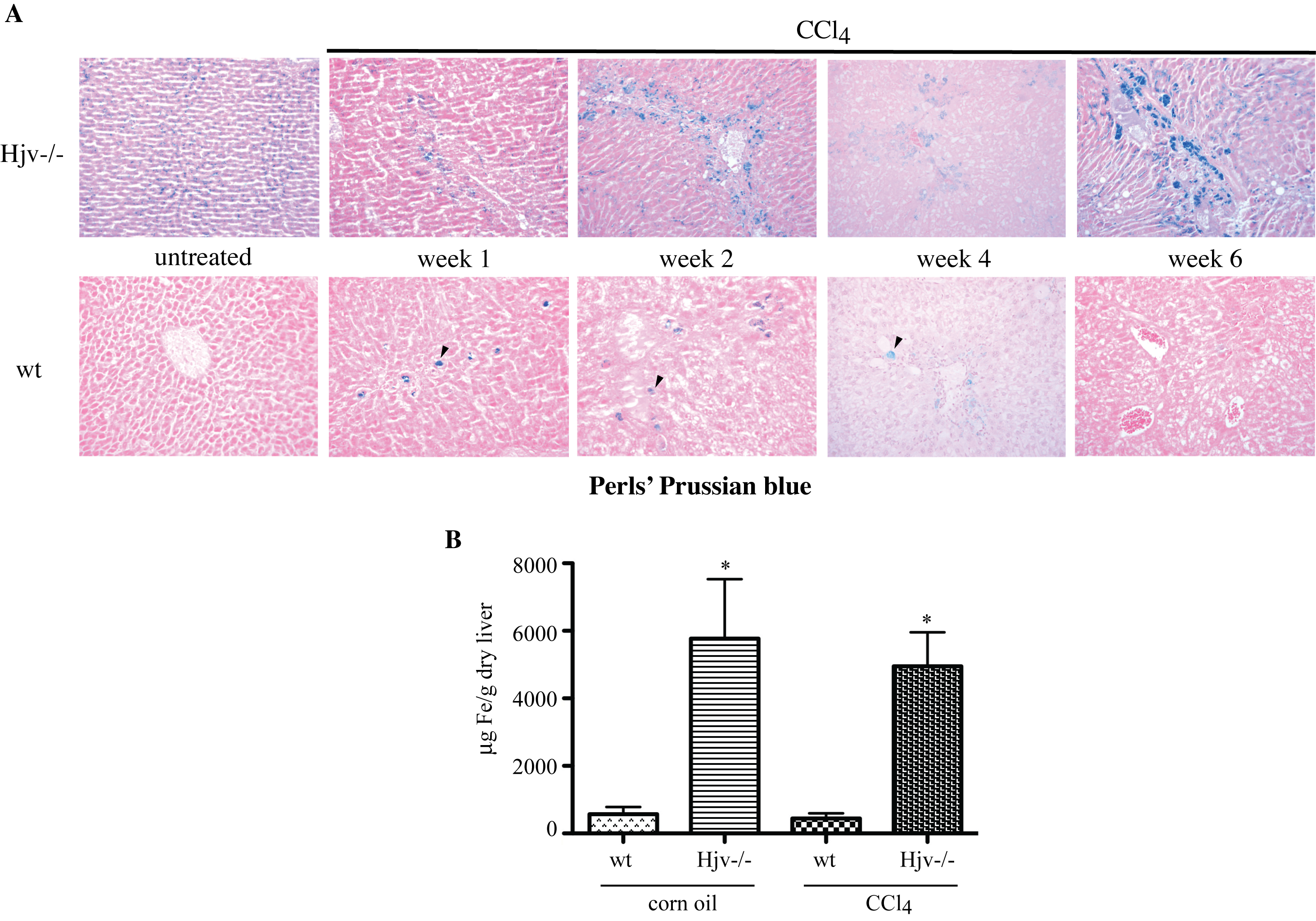

Supplement: Figure S2 — Hjv−/− mice exhibit severe iron overload in parenchymal cells, while the CCl4 treatment promotes iron retention within Kupffer cells. (A) Perl' s staining of liver sections from Hjv−/− and wt mice subjected to treatment with CCl4 for 1–6 weeks. Iron-loaded Kupffer cells are indicated with arrowheads. Original magnification 20x. (B) Quantification of hepatic non-heme iron by the ferrozine assay. *p<0.05 vs wt (Student's t test). (TIF) [file pone.0025138.s002.tif]

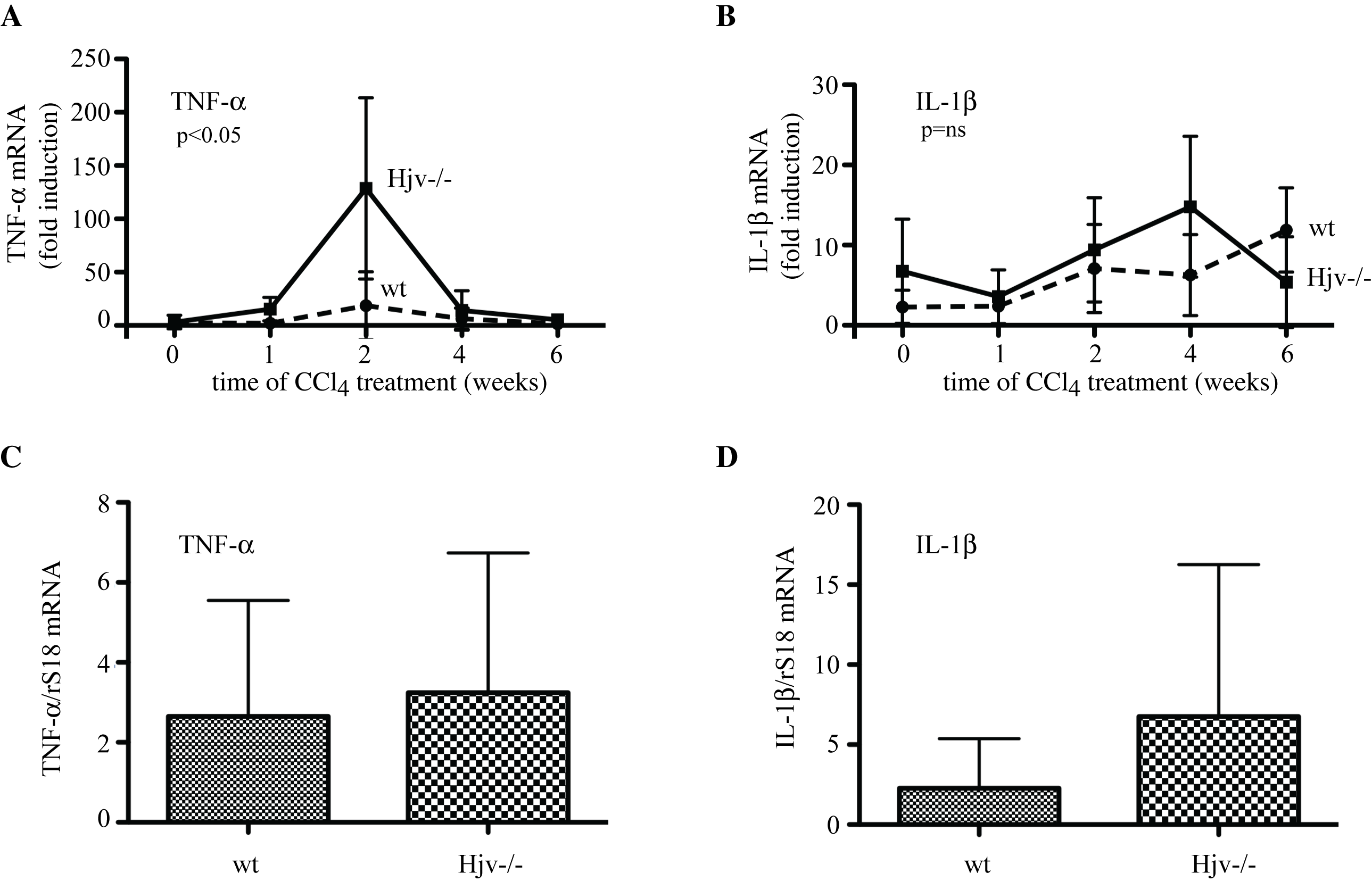

Supplement: Figure S3 — Expression of proinflammatory cytokines in Hjv−/− and wt mice. (A and B) The induction of TNF-α and IL-1β mRNAs corresponds to the ratios of respective values obtained from CCl4- and corn oil-treated animals. The p values refer to Hjv−/− vs wt and were obtained by the ANOVA test; ns = non-significant. (C and D) Comparative expression of TNF-α and IL-1β mRNAs in livers from naïve wt and Hjv−/− mice. (TIF) [file pone.0025138.s003.tif]

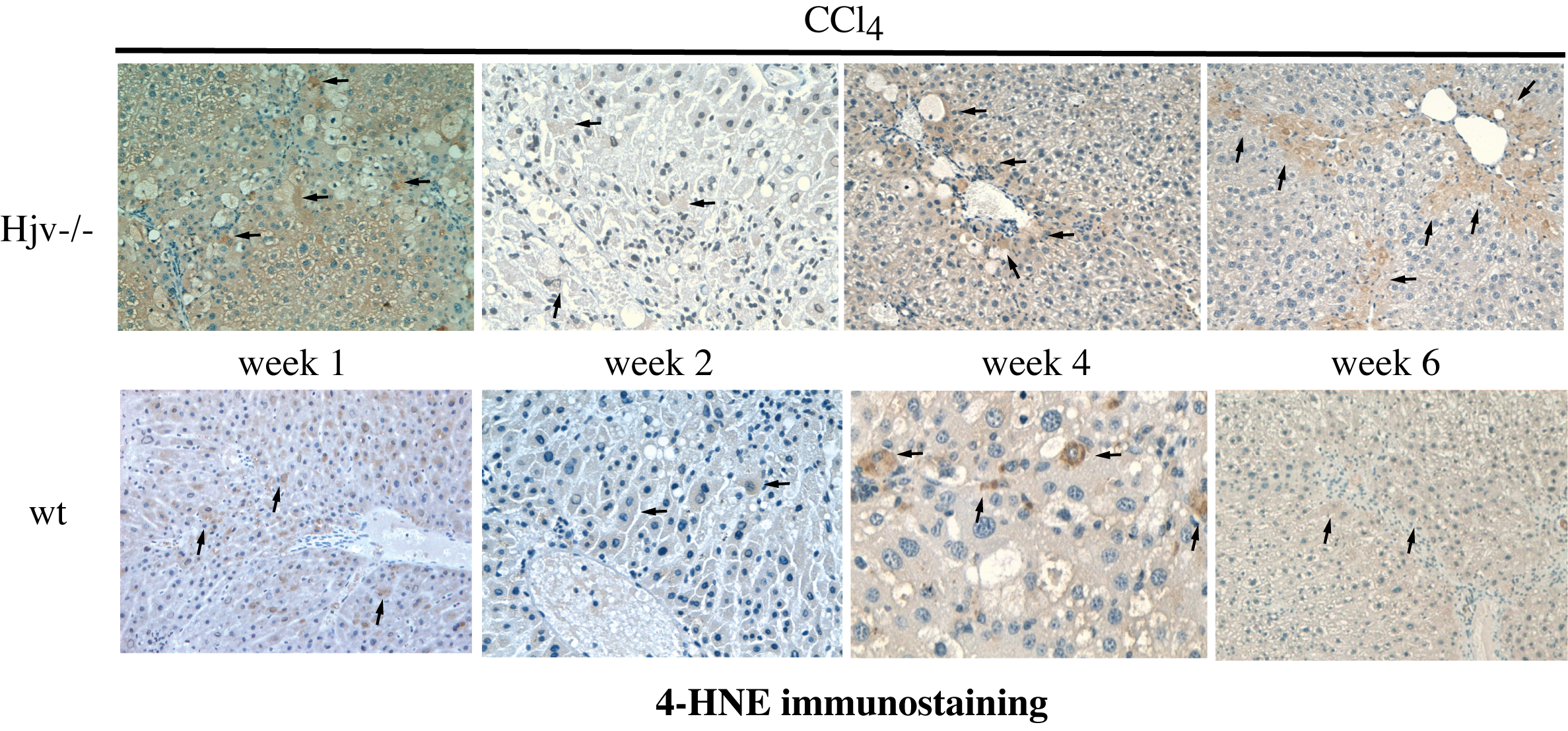

Supplement: Figure S4 — Immunohistochemical detection of 4-HNE (arrows) in livers of CCl4-treated Hjv-/- and wt mice. Original magnification 20x, except wt mice week 4 (40x). (TIF) [file pone.0025138.s004.tif]
